# ABO and Rh blood groups in patients with lupus and rheumatoid arthritis

**DOI:** 10.22088/cjim.12.4.568

**Published:** 2021

**Authors:** Amir Nik, Zahra Mirfeizi, Zahra Rezaieyazdi, Mandana khodashahi, Shahin Danevash, Mohammad Sobhan Sheikh Andalibi, Mahnaz Abbasi, Maryam Sahebari

**Affiliations:** 1 Rheumatic Diseases Research Center, Mashhad University of Medical Sciences, Mashhad, Iran; 2 Qazvin Metabolic Disease Research Center, Qazvin University of Medical Sciences, Qazvin, Iran

**Keywords:** Rheumatoid arthritis (RA), Systemic lupus erythematosus (SLE), ABO blood group, Rh blood group.

## Abstract

**Background:**

Systemic lupus erythematous (SLE) and rheumatoid arthritis (RA) are autoimmune diseases in which the antigen-antibody system plays an important role. As blood group and Rh are determined by the presence or absence of antigens on the surface of red blood cells (RBCs), we aimed to determine the distribution of ABO and Rh blood groups in SLE and RA patients and its association with disease manifestations.

**Methods:**

This short communication is based on a study that was conducted on 434 SLE and 828 RA patients. We evaluated the distribution of ABO and Rh blood groups in RA and SLE patients.

**Results:**

This study projected that in lupus patients, Coombs-positive autoimmune hemolytic anemia and arthritis were more common among the B blood type and Rh-positive group, respectively. Furthermore, there was no relation between ABO and Rh blood group and rheumatoid factor (RF) and anti-Cyclic Citrullinated Peptide (anti-CCP) seropositivity. Moreover, there was no difference in distribution of blood groups in RA and SLE patients.

**Conclusion:**

The higher frequency of blood group B in hemolytic anemia, and positive Rh in arthritis in lupus patients, develop the hypothesis of probable role of ABO blood group antigen in some manifestations of lupus.

ABO blood group system is based upon the presence or absence of glycoproteins A and/or B on the surface of red blood cells (RBCs) (1). The mentioned antigens, play an important role in immunologic responses. The presence or absence of D antigen is reported as Rh positive and Rh negative, respectively (2). Systemic lupus erythematous (SLE) is a chronic autoimmune disorder characterized by specific autoantibodies. Lupus can present with hemolytic anemia, leucopenia and thrombocytopenia, thrombotic thrombocytopenic purpura, and multiple organ involvement (3). Rheumatoid arthritis (RA) is also an autoimmune disease with autoantibody formation, symmetrical arthritis of small and large joints with non-specific hematologic manifestations (4). Several studies evaluated the role of blood groups in different diseases. A greater risk for thromboembolic and cardiovascular accidents has been presented in non-O blood types (5). Besides, raising the odds of severe P. falciparum infection have been reviewed among people with type O blood (6). The first report on correlation between the blood groups and rheumatic diseases by Cohen in 1963, demonstrated no significant heterogeneity in the results obtained from different diseases (7). Moreover, some studies showed the differences in blood group distribution among patients with different types of autoimmune diseases.

Although RA was more common in the patients with A blood type; SLE was more prevalent among the patients with type O blood. In addition, blood type AB was less observed in all diseases compared to the others. There was considerable difference in the distribution of Rh factor in rheumatic diseases. Çildağ et al. presented that different genetic predisposition was associated with higher incidence of different rheumatic diseases. They noted the distribution of ABO blood groups in the world is O>A>B>AB, whereas it is A>O>B>AB and Rh+>Rh- in Turkey (8). 

It has been proposed that identifying patients’ blood group with autoimmune hemolytic anemia, can be interfered with autoantibody (9). Misinterpretation of blood group in patients with lupus has been recorded, due to autoantibody production after antigenic stimulation (10).

Those interesting findings point out the possible interference between blood group production pathways and SLE-associated autoantibodies. As a result of showing hematologic manifestations by lupus, we aimed to study the distribution of blood group and Rh in lupus and its organ involvements. 

RA was also selected, due to the fact that it is also an autoimmune autoantibody formative disease without any specific hematologic manifestations.

## Methods

This is a short communication on the basis of a cross-sectional study which was conducted on 434 lupus and 828 RA patients, who were consecutively chosen from March 2014 to May 2017. The SLE and RA patients were diagnosed and classified by the 1997 American College of Rheumatology Revised Criteria, and 2010 ACR/EULAR Criteria, respectively (11,12). These patients were referred to the Rheumatic Diseases Research Center in Mashhad, and Qazvin Metabolic Disease Research Center in Qazvin. The recorded data included demographic features, clinical manifestations from the beginning stages of the disease until data collection, autoantibodies, blood group and Rh. 

Since accessing to the blood group information of the volunteer samples from general population of Iran with no bias was not possible during this research, we applied a secondary data. This information was retrieved from the latest, comprehensive study on this issue by Shahverdi et al. (13), in which the blood type distribution of the general population was: O>A>B>AB.

Data were analyzed using SPSS Version 11.0. The normality of data was assessed by applying the Kolmogorov-Smirnov test. Descriptive data were presented as mean (±SD) for normally distributed variables. 

Chi-square test was used to compare the qualitative variables between blood and Rh groups. Bonferroni correction was applied to minimize type I error. A p-value of less than 0.05 was considered statistically significant.  

Ethical approval for this study was obtained from the Ethics Committee of Mashhad University of Medical Sciences, Mashhad, Iran (No# 941268).

## Results

This study was conducted among 1262 (86.9% females, n: 1092) individuals, including 434 SLE (91.0% females, n: 395) and 828 RA (84.9% females, n: 703) patients.

**Lupus patients: **Average duration of disease was 6.8±6.02 years. The mean (±SD) of the age distribution was 34.3±11.5 years. Table-1 projects the distribution of clinical manifestations of lupus, among subtypes of ABO/Rh blood group. 

As shown in table 1, Coombs-positive autoimmune hemolytic anemia was significantly more frequent among the B blood group (P=0.03, χ2=4.66). Additionally, arthritis was more common in Rh-positive patients compared to negative ones (P=0.02, χ2=4.99). 

There was no significant difference between blood groups and other clinical manifestations in SLE patients. The most prevalent blood group and Rh in our lupus and RA patients were O+

**RA patients: **Average duration of disease was 5.7±5.6 years. The mean of the age distribution was 50.1±13.3 years. 75.6% of patients were anti-CCP positive and 66.4% were RF positive. No association was found between ABO blood group and anti-CCP (P=0.80, χ2=0.96) and RF (P=0.92, x2 =0.48). Furthermore, no evidence for association of Rh blood group, anti-CCP (P=0.50, x^2^ =0.017) and RF (P=0.52, χ2 =0.01) was recognized. 

It should be noted that there was no difference in distribution of ABO and Rh blood groups between RA and SLE patients (table 2).

As we mentioned in the method section, we used the latest secondary data about our national blood group distribution. We found that the blood group distribution of the general population was similar to our patients: O>A>B>AB.

**Table 1:** Distribution of clinical manifestation of lupus, among subtypes of blood groups and Rh of SLE patients

Blood group & Rh Organ Involvements	A N (%)	B N (%)	AB N (%)	O N (%)	*P*-value	Rh + N (%)	Rh – N (%)	*P*-value	Total Population N (%)
	126 (31.3)	98 (24.4)	31 (7.7)	147(36.6)	(χ2)	374(92.3)	31(7.7)	(χ2)	434(100)
Nephritis	25 (19.8)	28 (28)	6 (19.4)	43 (29.1)	0.25 (4.17)	89 (23.8)	13 (41.9)	0.02 (4.99)	102 (25.2)
Myositis	2 (1.6)	10 (2.5)	0.44 (0.85)	0 (0.0)	10 (2.7)	0.65 (1.83)	5 (3.4)	0 (0.0)	3 (3.0)
Vasculitis	20 (15.9)	52 (12.8)	0.41(0.3)	3(9.7)	49 (13.1)	0.14 (5.38)	15 (10.2)	1 (3.2)	16 (16.3)
Arthritis	61 (48.4)	193 (47.7)	0.02 (4.6)	9 (29.0)	184 (49.2)	0.54 (2.26)	76 (51.4)	13 (41.9)	43 (43)
Dermatitis	55 (43.7)	195 (48.1)	0.43 (0.16)	14 (45.2)	181 (48.4)	0.33 (1.85)	72 (48.6)	13 (41.9)	55 (55.0)
Carditis	9 (7.1)	32 (7.4)	0.40 (4.4)	3 (11.5)	27 (7.3)	0.61 (2.12)	9 (6.1)	4 (12.9)	8 (8.0)
CNS	12 (9.5)	50 (12.3)	0.72 (6.08)	43 (11.5)	7 (22.6)	0.49 (2.05)	19 (12.8)	6 (19.4)	13 (13.0)
Leukopenia	18 (14.3)	65 (16.0)	0.23 (1.01)	3 (9.7)	62 (16.6)	0.38 (3.05)	23 (15.5)	3 (9.7)	21 (21.0)
Thrombocytopenia	23 (18.3)	75 (18.5)	0.47 (0.12)	5 (16.1)	70 (18.7)	0.96 (0.27)	27 (18.2)	5 (16.1)	20 (20.0)
Lymphopenia	10 (7.9)	47 (11.6)	0.50 (0.12)	3 (9.7)	44 (11.8)	0.14 (5.34)	18 (12.2)	2 (6.5)	17 (17.0)
Hemolytic anemia	18 (14.3)	66 (16.1)	0.42 (0.59)	4 (12.9)	62 (16.3)	0.03 (8.63)	23 (15.5)	1 (3.2)	24 (24.0)
Positive ANA	119 (94.4)	368 (90.6)	0.55 (0.91)	28 (90.3)	340 (90.9)	0.32 (3.48)	134 (90.5)	27 (87.1)	88 (88.0)
Positive anti-dsDNA	77 (78.6)	240 (75.8)	0.26 (0.36)	20 (83.3)	220 (75.1)	0.49 (2.33)	87 (77.7)	19 (76.0)	57 (69.5)

**Table 2:** Distribution of ABO and Rh blood group among RA and SLE patients

**Variable**		**SLE group**	**RA group**	**χ2**	P-value*
		**No (%)**	**No (%)**		
**Blood group**	A	126 (31.2)	244 (30.1)	3.37	0.33
	B	100 (24.8)	216 (26.7)		
	AB	31 (7.7)	83 (10.3)		
	O	147 (36.4)	266 (32.9)		
Rh	Rh +	374 (92.3)	701 (92.4)	0.00	0.99
	Rh -	31 (7.7)	58 (7.6)		

*Chi-square test was used to compare the distribution of ABO and Rh blood groups between RA and SLE patients.

## Discussion

This study suggested a higher frequency of Coombs-positive autoimmune hemolytic anemia in B blood group and arthritis in Rh-positive lupus patients. Besides, there was no difference in the distribution of blood types and Rh between RA and lupus. According to the existing documents, the distribution of ABO/Rh blood types was the same as our national distribution.

As indicated above, the first report belongs to 57 years ago, and the following article determined the blood groups of 99 patients with some varieties of articular disease; (31 cases with RA, 31 individuals with spondylitis, 15 patients with gout, 9 with disseminated lupus erythematosus, 6 with familial Mediterranean fever, and 7 with other diseases). Statistical analysis demonstrated no significant heterogeneity among the results obtained from different diseases (7). The last comprehensive study on this topic was conducted by Çildağ et al. (2017) which presented a difference in the frequency and distribution of blood groups in RA patients, among different nationalities and races. The distribution of ABO blood groups distribution in the world is O>A>B>AB, whereas it is A>O>B>AB and Rh+>Rh- in Turkey (8). These results are different from our study. Since accessing to the blood group information of the volunteer samples from general population of Iran with no bias was not possible during this research, we applied a secondary data from the latest, comprehensive study by Shahverdi et al. (13), in which the blood type distribution of the general population was: O>A>B>AB.

The first comprehensive report on the prevalence of various RBC antigens and phenotypes of diverse blood groups in general population of Iran was published by Shahverdi and et al. (2016). Blood type O was the most prevalent followed by A, B, and AB groups. According to several studies, this is similar to blood group distribution in the United States (13). These results are in line with the results described in the present study. Karadağ A. conducted a study to compare the distribution of blood groups in inflammatory rheumatic diseases and healthy subjects. He reported the A blood type was more prevalent in patients with inflammatory rheumatic disease and in the healthy subjects, followed by O, B, and AB blood groups, respectively. However, there was no significant difference between the ABO groups in terms of distribution (p>0.05). The Rh positive blood group was more prevalent in both groups compared to Rh negative, but there was a statistically significant difference in the Rh blood group distribution among the two groups (14). 

Tamega et al. found discoid lupus erythematous is more severe in A blood group patients (15). Çildağ et al. suggested there was a significant difference in the distribution of blood groups in rheumatic diseases. Spondyloarthropathies, vasculitis, Behçet’s, and RA were more common in A blood groups; Familial Mediterranean fever, lupus, and Sjogren were more common in O blood group. In addition, AB blood type was less common in those autoimmune diseases. While, they suggested that positive Rh is more prevalent in autoimmune diseases (8). Mosaca et al. reported hemolytic anemia as an early presentation of lupus, is valuable in distinguishing SLE from its mimickers (16). Some studies pointed to a possible association between some autoantibodies and Rh blood group. Many of these autoantibodies are specific for Rh antigens, and they commonly react weakly to Rh-negative compared to Rh-positive. These autoantibodies may be nonreactive only with specific Rh and D−negative RBCs (17). In our study, SLE patients with Rh-positive showed significantly higher presentation of arthritis. It may be useful to predict a less prevalence of arthritis in Rh-negative patients. There was no evidence for any influence of blood group and Rh in positive autoantibodies in RA patients. Additionally, blood type and Rh had no different distribution in RA and lupus patients.

Hemolytic anemia and arthritis were more frequent in B blood group and Rh-positive lupus patients, respectively. There was no relationship between blood, Rh types and rheumatoid factor (RF) and anti-CCP. There was not any difference in blood groups and Rh distribution in RA and lupus. There are few articles regarding this topic, and this study is one of the first articles investigated any link between those variables. Because of our large sample size and also existence of volunteer bias in the information of blood transfusion center, we were unable to compare our findings with a matched control group, simultaneously. Therefore, we had a noticeable limitation in this regard. Thus, further studies can better assess the role of blood group-related autoantibodies in different presentations of SLE and RA.
